# Advanced Renal Pelvic Carcinoma Revealed after Treatment of a Staghorn Calculus by Endoscopic Combined Intrarenal Surgery

**DOI:** 10.1155/2020/9703479

**Published:** 2020-05-29

**Authors:** Ichiro Tsuboi, Yuki Maruyama, Motoo Araki, Nobuyoshi Ando, Yasuhiro Nishiyama, Ryoji Arata, Noriaki Ono

**Affiliations:** ^1^Department of Urology, Kochi Health Sciences Center, 2125-1 Ike, Kochi city, Kochi 781-8555, Japan; ^2^Okayama Urological Research Group (OURG), 2-5-1, Shikata-cho, Kita-ku, Okayama 700-8558, Japan; ^3^Department of Urology, Okayama University Graduate School of Medicine, Dentistry and Pharmaceutical Sciences, 2-5-1 Shikata-cho, Kita-ku, Okayama 700-8558, Japan

## Abstract

Renal pelvis carcinoma associated with staghorn calculus is a clinically rare condition. A 66-year-old man presented with flank pain due to an 8 cm complete staghorn calculus. We performed three lithotomies using endoscopic combined intrarenal surgery and carried out intraoperative biopsy. Histopathological examinations revealed a keratinized lesion. One month later, contrast-enhanced computed tomography showed an advanced renal pelvis carcinoma. These findings demonstrate that even an intraoperative biopsy may be insufficient to diagnose a renal pelvis carcinoma associated with a staghorn calculus. The possibility of RPCa developing when treating a long-standing staghorn calculus should therefore be kept in mind.

## 1. Introduction

Renal pelvic carcinoma (RPCa) associated with a staghorn calculus is a rare clinical condition, although it is well known that chronic irritation, inflammation, and infection from a long-standing renal stone can cause a RPCa [1, 2]. RPCa with a squamous cell carcinoma is an especially rare occurrence and accounts for only 0.5 to 0.8% of malignant renal tumors [3]. The preoperative diagnosis of urothelial carcinoma in patients with a staghorn calculus remains difficult even if computed tomography (CT) and urine cytology are performed [2]. Although intraoperative biopsies and urine cytology were not enough to diagnose due to the inflammation by the stone, they give us a chance of thinking about a possibility of malignancy. To our knowledge, there are no reports of RPCa being identified quickly within one month after endoscopic combined intrarenal surgery (ECIRS). We report a case of RPCa with a staghorn calculus, which possibly was disseminated by ECIRS.

## 2. Case Presentation

**Figure 1:**
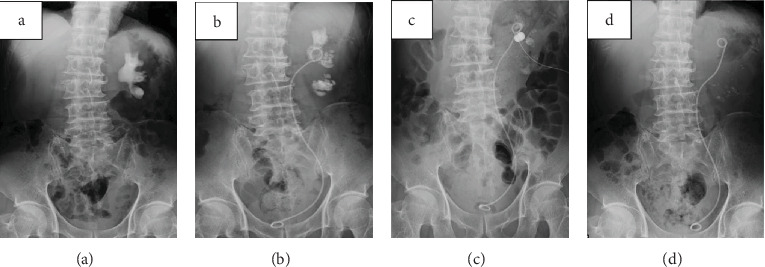
(a) A complete staghorn calculus of the left kidney (about 8 cm). (b) After the 1st ECIRS, a renal stone in the pelvis was removed. (c) After the 2nd ECIRS, most of the renal stone was removed. (d) After the last ECIRS, a stone-free state was achieved.

**Figure 2:**
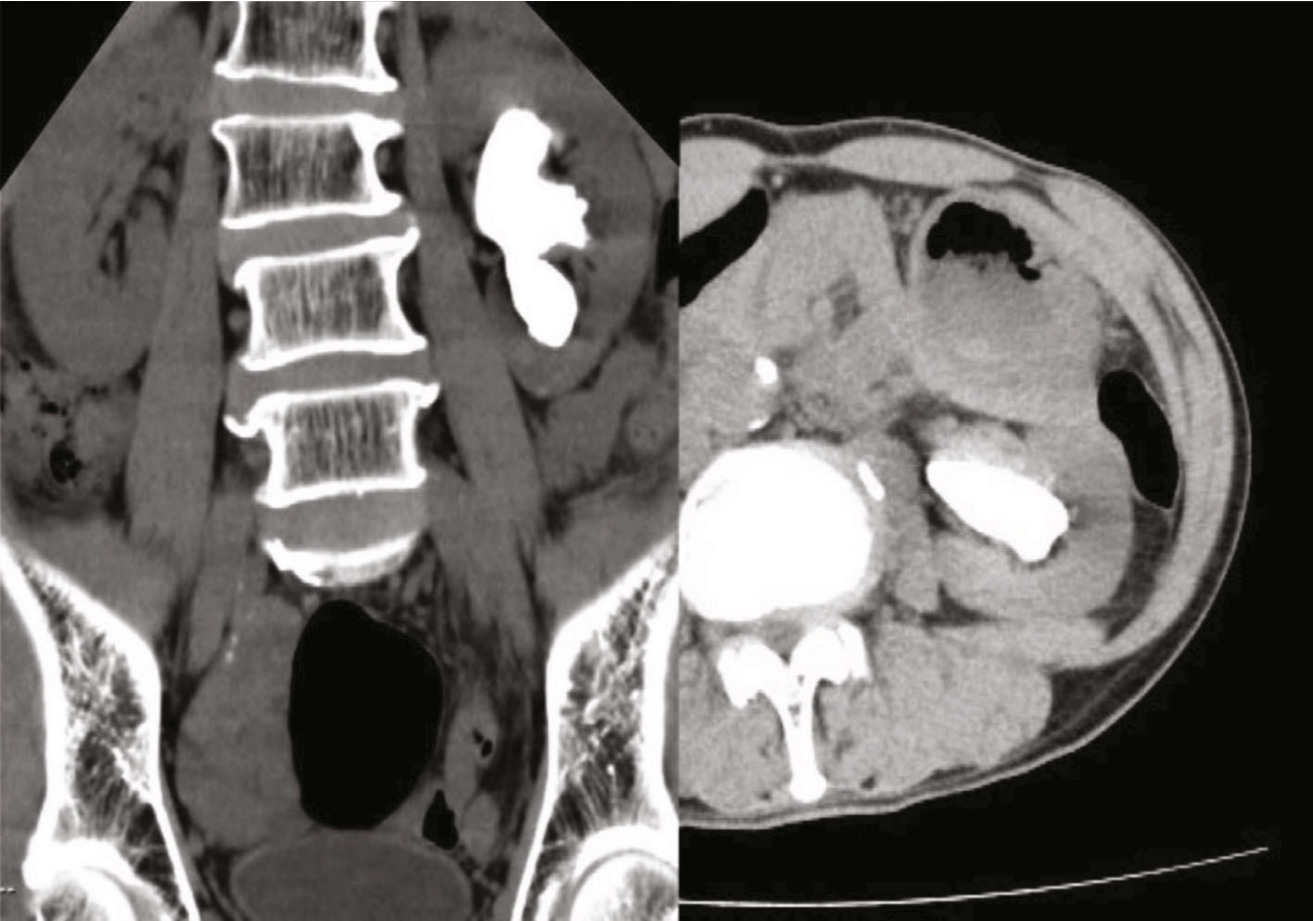
A complete staghorn calculus of the left kidney. The major diameter was about 8 cm. We could not identify the renal pelvic carcinoma retrospectively.

**Figure 3:**
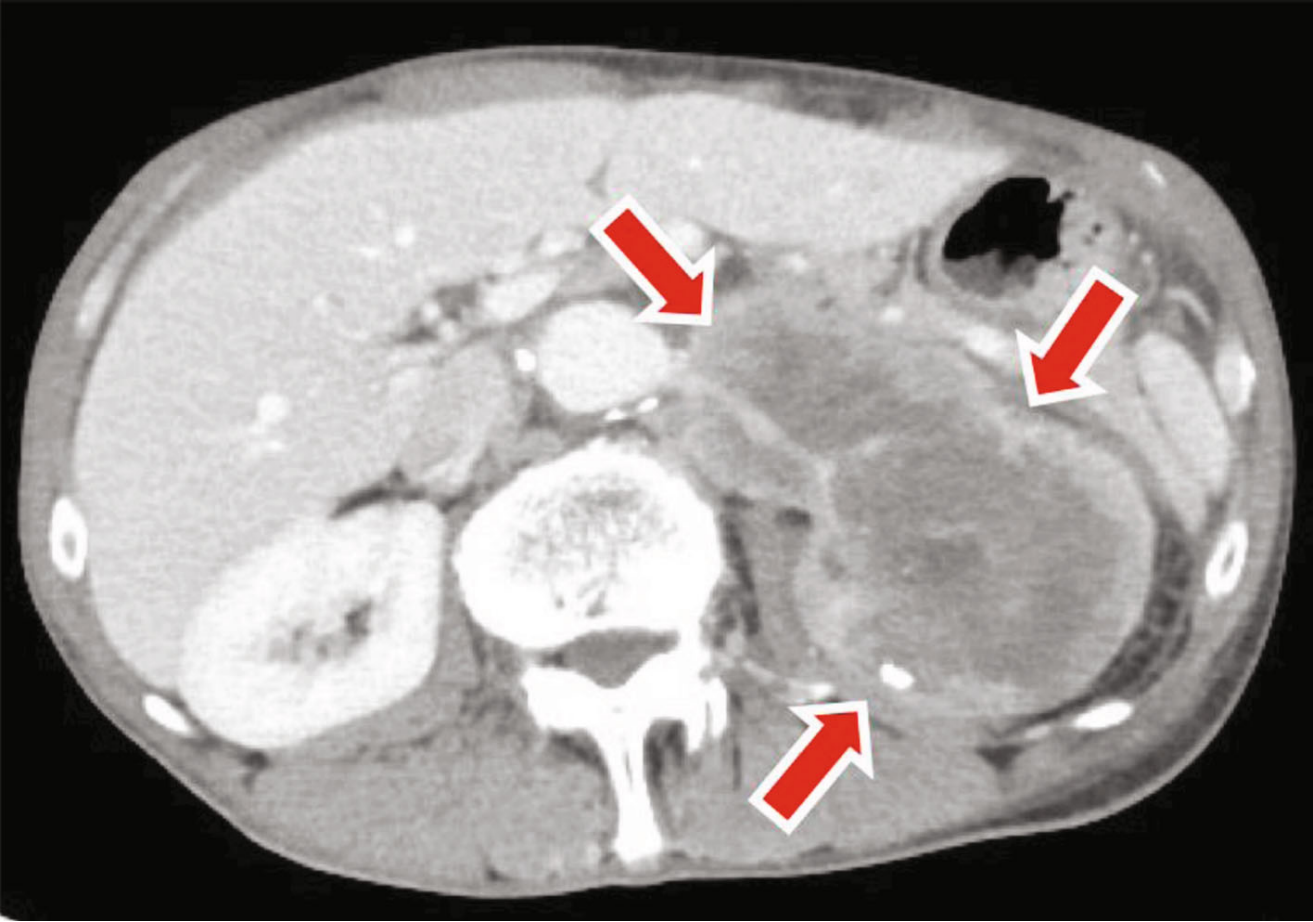
The renal tumor invaded the renal parenchymal and expanded about 10 cm. Lymph nodes can be seen expanded around the renal artery (red arrows). The clinical stage was classified as T4N1M0.

**Figure 4:**
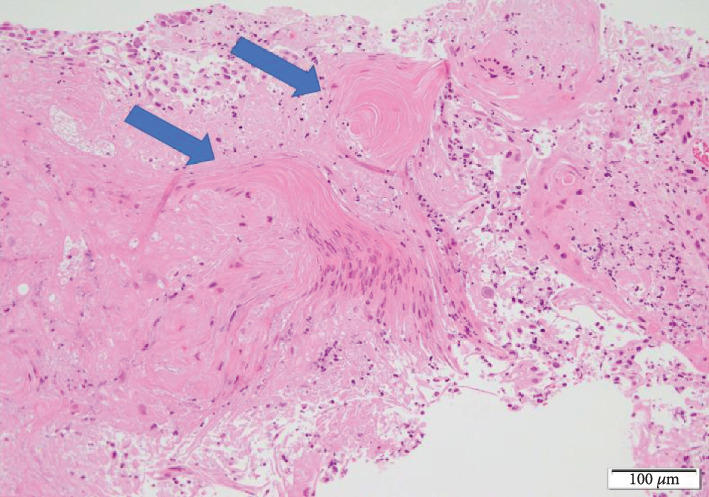
Urothelial carcinoma with squamous differentiation. Stratified squamous epithelium and keratinization of the lesion can be seen in some areas.

A 66-year-old man had been aware of left flank pain for 6 months prior to visiting his family doctor. The doctor carried out an X-ray of the kidney, ureter, and bladder and made a diagnosis of a staghorn calculus in the left kidney (Figure 1(a)). The patient was referred to our office for further examination and treatment. His previous medical history was a urinary tract stone 20 years ago. We performed no contrast-enhanced CT of the abdominal and pelvis, which revealed an 8 cm renal stone in the left renal pelvis (Figure 2). Urine tests showed hematuria and pyuria, but no bacteriuria, while the results of blood tests were unremarkable with a serum creatinine concentration of 0.95 mg/dL. We diagnosed a staghorn calculus in the functioning left kidney and attempted to perform an ECIRS, first establishing hydronephrosis using an occlusion catheter to puncture in order to make a percutaneous trocar nephrostomy easy. However, we were unable to insert the trocar, despite the left kidney being punctured three times. A lithotripsy was only performed. Part of the left renal stone still existed, especially in the inferior calyx of the kidney (Figure 1(b)). Analysis showed the stone consisted of calcium phosphate. ECIRS was performed one month later, and we were able to insert the trocar at this time and carry out the procedure as planned (Figure 1(c)). Analysis of the stone showed it consisted of calcium oxalate (90%) and calcium phosphate (10%). At this time, we realized that the white fuzzy tissue was different from normal renal pelvic mucosa and therefore carried out urine cytology and a single targeted biopsy of the renal pelvic mucosa. Urine cytology identified squamous cells classified as class III. Histopathological examination showed a keratinized lesion and stratified squamous epithelium with atypical cells, although we were unable to determine whether these were malignant changes. ECIRS was performed and allowed the majority of the kidney stone to be removed (Figure 1(d)). One month after the last surgery, the patient complained of persistent left flank pain, with contrast-enhanced CT showing that almost all of the left kidney was invaded and replaced by a tumor. The tumor had expanded to around the left renal artery although there was no metastatic lesion (Figure 3). We performed a left renal biopsy because we suspected a renal cell carcinoma or RPCa. Histological examination revealed an invasive urothelial carcinoma with squamous differentiation and a clinical stage of T4N0M0 (Figure 4). We then performed two courses of neoadjuvant chemotherapy (cisplatin and gemcitabine). After two courses of chemotherapy, CT showed stable disease. We judged that this chemotherapy was ineffective, and it is difficult to operate radical nephrectomy in this case. We started to administer pembrolizumab. After we performed 7 courses, CT showed progress disease. We stopped to administer pembrolizumab and started palliative treatment.

## 3. Discussion

RPCa with a staghorn calculus is a rare case and difficult to diagnose preoperatively, although intraoperative urine cytology and biopsy may indicate the possibility of a malignancy [2]. It was highly possible in our case that ECIRS disseminated the RPCa. This is the case of the advanced RPCa, which was revealed in one month after treatment of staghorn calculus by ECIRS.

A RPCa with staghorn calculus is a clinically rare case, although we, urologists, well know that a long-standing renal stone may cause malignancy because of chronic irritation, inflammation, and infection. Indeed, a retrospective study of more than 500 percutaneous nephrolithotomies (PCNL) showed three patients had RPCa that had not been diagnosed preoperatively. One patient who had a biopsy taken from suspicious looking tissue in the renal pelvis during PCNL was diagnosed with RPCa [2]. That report described difficulty diagnosing the RPCa by CT and urine cytology preoperatively. Our case showed that even intraoperative cytology and a biopsy did not confirm the diagnosis and was characterized by very early identification of the RPCa, leading us to suspect ECIRS had disseminated the tumor. Two cases of RPCa after PCNL have been reported although in these cases, PCNL had been performed 2 and 16 years earlier [4]. These cases should be distinguished from our case because we consider that there was no RPCa present when the PCNL was performed. On the other hand, Kim et al. reported a similar case of a 54-year-old woman who presented with a skin mass that surrounded the muscle tissue of the right flank. The patient had been treated for a right staghorn calculus by PCNL three months earlier. Histological examination of the skin mass showed an invasive well-differentiated squamous cell carcinoma. The PCNL had spread to surrounding tissues via the percutaneous nephrostomy track [5]. To our knowledge, there are only two similar reports (3 cases) in which RPCa appears to have been disseminated by PCNL (Table 1).

Preoperative urine cytology and CT should be performed to prevent spread of RPCa, even if there is the possibility of the false negative. In addition, we perform intraoperative urine cytology and a biopsy when we identify an abnormal renal pelvis mucosa. Because RPCa tends to be aggressive and invasive to renal tissues, it is likely a targeted and systematic renal biopsy during PCNL may contribute to an early diagnosis.

## 4. Conclusion

RPCa with a staghorn calculus is difficult to diagnose preoperatively. An intraoperative renal pelvis mucosa biopsy is not always useful to confirm the diagnosis of RPCa. The possibility of RPCa developing when treating a long-standing staghorn calculus should therefore be kept in mind.

## Figures and Tables

**Table 1:** Three cases of renal pelvic carcinoma with staghorn calculus, which was revealed after percutaneous nephrolithotomy.

Authors [references]	Age	Sex	Duration after PNL	Location of spread	Histological examination	Treatment	Follow-up/survival
Kim et al. [5]	54	Female	1 mth	Nephrostomy of PCNL	SCC	Resection of the skin mass Radical nephrectomy	12 mth/alive
Katz et al. [2]	50	Male	2 wk	Lower pole of the kidney	UC	Radical nephrectomy Chemotherapy	19 mth/death
Katz et al. [2]	65	Female	Inoperative	Diaphragm	UC with sarcomatoid	No	2 mth/death
Tsuboi et al. [present report]	66	Male	1 mth	Left kidney Para-aortic lymph node	UC with squamous differentiation	Chemotherapy	3 mth/alive

## Abbreviations

CT: Computed tomography
ECIRS: Endoscopic combined intrarenal surgery
PCNL: Percutaneous nephrolithotomy
RPCa: Renal pelvic carcinoma.
